# Exosomes from miR-23 Overexpressing Stromal Cells Suppress M1 Macrophage and Inhibit Calcium Oxalate Deposition in Hyperoxaluria Rat Model

**DOI:** 10.1155/2023/2883623

**Published:** 2023-11-16

**Authors:** Zhang Yifan, Zhang Shengli, Wang Min, Cheng Wenjie, Sun Yi, Xu Luwei, Jia Ruipeng

**Affiliations:** Department of Urology, Nanjing First Hospital, Nanjing Medical University, 68 Changle Road, Nanjing 210006, China

## Abstract

**Purpose:**

To investigate whether ADSC-derived miR-23-enriched exosomes could protect against calcium oxalate stone formation in a hyperoxaluria rat model.

**Methods:**

An ethylene glycol- (EG-) induced hyperoxaluria rat model and an in vitro model of COM-induced HK-2 cells coculturing with RAW264.7 cells were established to explore the protective mechanisms of ADSC-derived miR-23-enriched exosomes.

**Results:**

The results showed that treatment with miR-23-enriched exosomes from ADSCs protected EG-induced hyperoxaluria rats, and cell experiments confirmed that coculturing with miR-23-enriched exosomes alleviated COM-induced cell autophagy. Overexpressed miR-23 suppressed M1 macrophage polarization by inhibiting IRF1 expression. Furthermore, the predicted binding site between the IRF1 messenger RNA 3′-untranslated region (3′-UTR) and miR-23 was confirmed by the dual-luciferase reporter assay.

**Conclusion:**

In conclusion, our research gave the first evidence that ADSC-derived miR-23-enriched exosomes affected the polarization of M1 macrophages by directly inhibiting IRF1 and protecting against calcium oxalate stone formation in a hyperoxaluria rat model.

## 1. Introduction

Kidney stone is one of the most common urological diseases which are closely related to genetic, environmental, metabolic, and other factors [1]. According to their chemical composition, kidney stones can be categorized into calcium-containing stones, infectious stones, cystine stones, uric acid stones, and drug stones. Among them, calcium oxalate (CaOx) stone is the most common component, accounting for about 70-80% of all stone types. The recurrence rate of CaOx stones is 50% in 5-10 years and up to 70%-80% in the recent 20 years [2]. The high recurrence of CaOx stones has significantly threatened human health and caused great economic losses.

Many reports demonstrated that inflammation-induced injury to renal tubular epithelial cells could change the structure and polarity of the cell membrane surface, providing adhesion sites for CaOx crystals and promoting the formation of CaOx stones [3]. Studies have identified that after exposure to CaOx crystals, MCP-1 expression was significantly increased in renal tubular epithelial cells (RTECs), and macrophage recruitment in the renal interstitium was also increased [4]. Macrophages are expected to differentiate into different phenotypes known as polarization, including classical activation (M1) and selective activation (M2), which may play proinflammatory and anti-inflammatory roles, respectively [5]. It has been illustrated that macrophages and their M1/M2 polarization states are central to the pathogenesis of CaOx stone formation. Taguchi et al. found that CaOx crystals could promote the M1-type polarization of macrophages, damage renal tubular epithelial cells, and contribute to the development of CaOx crystal deposition [6]. Singhto et al. revealed that M2-type macrophages could endocytose CaOx crystals and enhance the antiadhesion ability to protect renal tubular epithelial cells [7].

Interferon regulatory factor 1 (IRF1) is a member of the IRF family, which can encode two IRF1 protein isoforms. IRF1 has been verified to participate in various physiological and pathological processes, such as growth, differentiation, cell apoptosis, tumor initiation, inflammation, and viral infections [8]. Besides, IRF1 can also increase 8-fold in M1 macrophages induced by IFN-*γ* and lipopolysaccharide (LPS) in comparison with M0 and M2 macrophages, indicating that IRF1 may serve as a vital role in modulating M1 macrophage polarization [9]. Additionally, emerging evidence has proved that several miRNAs can regulate the expression of IRF1 [10]. There is evidence that miR-23 can modulate the polarization of M1 macrophages through suppressing IRF1 expression, suggesting that the expression of miR-23 may be critical to regulating renal tubular inflammation and CaOx formation [11].

Adipose-derived stem cells (ADSCs) are characterized by facile cell harvesting, ease of in vitro culture, and convenient acquisition [12–14]. Furthermore, unlike polymeric nanoparticles and liposomes, the exosomes derived from ADSCs can avoid immunoreaction and endosomal-lysosomal degradation [15] and have the potential to be ideal for gene-drug delivery [16]. In the present study, ADSC-derived miR-23-enriched exosomes could suppress M1 macrophage polarization in CaOx stone formation by inhibiting IRF1 expression. In this study, we aimed to explore the role and potential mechanism of exosomes derived from miR-23-overexpressing ADSCs in the treatment effects on CaOx stones.

## 2. Methods

### 2.1. Animal Experiment

In total, 30 male SD rats (weighing 100.0 ± 5.1 g) were housed in the experimental animal center of Nanjing Medical University (SCXK 2008-0004) under standard conditions. Throughout the trial, deionized distilled water was available to all rats without restriction. All the rats were fed with growth maintenance feed and purified water. This study was approved by the Veterinary Directorate of Nanjing Medical University according to Chinese law and the Council Directive of the Asian Community. On day 7, ketamine (set the concentration to 100 mg/kg) was used to anesthetize all rats, and the specimens were stored at -20°C until measurements were conducted.

### 2.2. Hyperoxaluria Rat Model

The SD rats were randomly divided into control, ethylene glycol (EG) (COM), EG + exosomes (COM + ADSC-exo), and EG + miR-exosome (COM + ADSC-miR-exo) groups. Control group rats had free access to tap water. The hyperoxaluria rat model was induced by allowing rats in the EG group free access to drinking water containing 1% EG. We isolated exosomes in 200 *μ*L PBS and administered them by intravenous injection daily for 4 weeks for the exo groups, whereas control rats received an equal volume of normal saline.

### 2.3. Renal Histology

All the kidney specimens were fixed with 4% paraformaldehyde and embedded in paraffin after 48 hours. Then, the tissues were sectioned into 4 *μ*m thick, followed by HE staining, and examined under a light microscope (Olympus BX40, Tokyo, Japan). Using a Nikon TE2000U inverted microscope and the program Meta-Morph, some were stained with hematoxylin and eosin and then scanned using polarization microscopy (Scion Inc, USA). With the use of an EM 410 transmission electron microscope, an ultrastructural investigation was carried out (Philips; Eindhoven, Netherlands). A monoclonal antibody (F4/80; clone BM8, Abcam, # 16911) that is directed against macrophages and monocytes was utilized in an avidin-biotin immunoperoxidase procedure.

### 2.4. Cell Culture

Human kidney-2 (HK-2) cells were obtained from ATCC (American Type Culture Collection) and employed as the experimental model. The HK-2 cells were cultured in Eagle's minimum essential medium and suppled with 10% fetal bovine serum and 1% penicillin-streptomycin. The cells were planted in six-well plates or T-75 flasks for the tests at a density of 2 × 10^5^ cells/mL. The cells were then treated with certain conditions. Platinum was sputter-coated onto the samples, which were then investigated using a JEOL JSM-6500 TFE-SEM at a 10 keV accelerating voltage.

### 2.5. The Culture of ADSCs

The adipose tissues used in this study were obtained from dead SD rats. After resection, the tissues were cleaned in PBS and cut into 1 mm square pieces. Then, they were digested with collagenase, followed by centrifuging at 4000 g for 5 minutes. The resulting cell pellet was suspended in Dulbecco's modified Eagle's medium (DMEM) containing 10% FBS, 100 U/mL penicillin, 100 g/mL streptomycin, and 2 mmol/L L-glutamine. The mixture was then cultivated for 48 hours at 38°C in a controlled environment with 5% CO_2_. Cells were taken out and placed into brand-new culture media, which was then replaced every three days. Cells were passaged and utilized at passing there when they were around 90% confluent. To validate the identification of ADSCs, cells were treated with conjugated monoclonal antibodies against CD29, CD44, CD90, CD105, CD31, and CD45, while isotype-identical antibodies (PharMingen) were utilized as controls. Following 1% paraformaldehyde fixation, ADSCs were analyzed quantitatively using a FACSCalibur Flow Cytometer from BD Biosciences and FlowJo software. For each sample, fluorescence intensities were measured logarithmically for 1 × 10^4^–2 × 10^4^ cells.

### 2.6. Isolation and Identification of Exosomes

miR-23 mimics and corresponding negative controls were transfected into ADSCs at a concentration of 20 nmol/L using Lipofectamine 3000 (Invitrogen Life Technologies). ADSCs were gathered 48 hours after transfection to examine miR-23 expression. At 80%-90% confluence, transfected ADSCs were washed with PBS before being cultivated in FBS-free microvascular endothelial cell growth medium-2 (EGM-2MV). The ADSCs were then supplemented with 1 serum replacement solution (PeproTech) for 24 hours. The ADSCs were centrifuged at 300 × g for 10 minutes and 2000 × g for 10 minutes to remove dead cells and debris. Ten milliliters of culture supernatant was collected by a high-speed cryopreservation centrifuge at 100,000 × g for 15 minutes at 4°C. Impurities were removed from the supernatant; the supernatant was transferred to another sterilized 15 mL centrifuge tube and 5 mL of ExoQuick-TC reagent (System Biosciences), followed by resuspending in nuclease-free water and then incubated overnight in a refrigerator at 4°C. Then, the mixture was centrifuged at 4°C at 1500 g for 30 minutes. White or beige precipitation at the bottom of the centrifuge tube was visible, and the supernatant was transferred to another tube for later experiments. Then, 100 *μ*L of PBS or sterile water was added after heavy suspension precipitation, and the samples were stored in the refrigerator at −80°C. The total RNA was extracted using TRIzol LS (Invitrogen), and the protein was isolated by the Exosomal Protein Extraction kit (Invitrogen), respectively. Exosomes were either employed right away for research or were kept frozen at -180°C. The diameters of isolated exosomes were measured by a NanoSight LM10 (Malvern Instruments) nanoparticle tracking system. The amounts of CD63 and TSG101 proteins were assessed by western blotting. The BCA test kit (Beyotime, Nantong, China) was utilized to measure the amount of protein in exosomes. The ultrastructure of the vesicles was examined using transmission electron microscopy (TEM) with a Libra 120 apparatus from Zeiss.

### 2.7. Cell Transfection

The miR-23 mimics or corresponding NC were bought from GenePharma (Shanghai, China). For transfection, the ADSCs were transfected with miR-23 mimic or NC at a concentration of 50 nmol/L through using Lipofectamine 3000 (Invitrogen) according to the manufacturer's instructions. The cells were used to analyze the expression of miR-23 in the following experiments after 48 hours of transfection.

### 2.8. Western Blot Analysis

The concentration of protein was measured by the bicinchoninic acid kit (Beyotime), and then approximately 20 mg of proteins was treated with 10% SDS-page, and then the proteins were transferred onto a nitrocellulose membrane and blocked with 5% nonfat dry milk at room temperature for 1 h. Following this, the membranes were incubated with primary antibodies overnight at 4°C. iNOS (ab178945, Abcam, 1 : 1000 dilution), TNF-*α* (ab307164, Abcam, 1 : 1000 dilution), and ARG1 (ab203490, Abcam, 1 : 1000 dilution) in this work. The membranes were then incubated with the proper secondary antibodies after being washed in TBST for 30 minutes. A commercially available enhanced chemiluminescence kit (Amersham-Pharmacia Biotech) and Kodak film were used to detect the bound antibodies.

### 2.9. Immunohistochemistry

Formalin-fixed, paraffin-embedded 4 *μ*m sections were utilized to conduct immunohistochemical stains. Sections were rehydrated, and hot citrate was used to extract the antigens. Tissue slices were exposed successively to the primary antibody, horseradish peroxidase-coupled secondary antibodies, and blocking buffer after incubation (Vectastain Elite; Vector Laboratories, Peterborough, UK). With the use of a DAB Peroxidase Substrate Kit (Vector Laboratories), the signals were created. Representative photos were shown, along with at least three more iterations of each immunohistochemistry analysis.

### 2.10. RNA Extraction and RT-PCR

Total RNA was isolated from tissues, cells, and exosomes through using TRIzol in accordance with the manufacturer's instructions, and complementary DNA (cDNA) was produced by using SuperScript reverse transcriptase and oligo dT18 primers from 1 g of total RNA. One liter of first-strand cDNA amplification product served as the template for conventional PCR. The RT-PCR was conducted by using Taq DNA polymerase (TaKaRa Bio), and the procedure was set as follows: 30 cycles of 94°C for 30 seconds, 55°C for 30 seconds, and 72°C for 30 seconds. The 2CT approach was used to calculate relative expression levels.

### 2.11. Luciferase Reporter Assay

GenePharma Corporation created a recombinant pmirGLO plasmid that has a binding sequence or a mutant sequence. Using Thermo Fisher Scientific's Lipofectamine 2000 reagent, 293 T cells were cotransfected with miR-23 mimics or NC with IRF1 wild-type plasmid (WT) or mutant-type plasmid (MUT). The Dual-Luciferase Reporter Assay System (Promega, Madison, WI) was utilized to measure the luciferase activity in accordance with the manufacturer's instructions.

### 2.12. Statistical Analysis

The statistical evaluations were all conducted by using SPSS (version 19.0). Frequencies were used to represent discrete variables, and the Pearson 2 test was used to assess differences. Continuous variables were summarized as the mean standard deviation (SD), and *t*-tests or analysis of variance were used to determine the significance of the data for groups larger than two. A *P* value of 0.05 or less was regarded as significant.

## 3. Results

### 3.1. The Construction of ADSC-Derived miR-23-Enriched Exosomes

**Figure 1:**
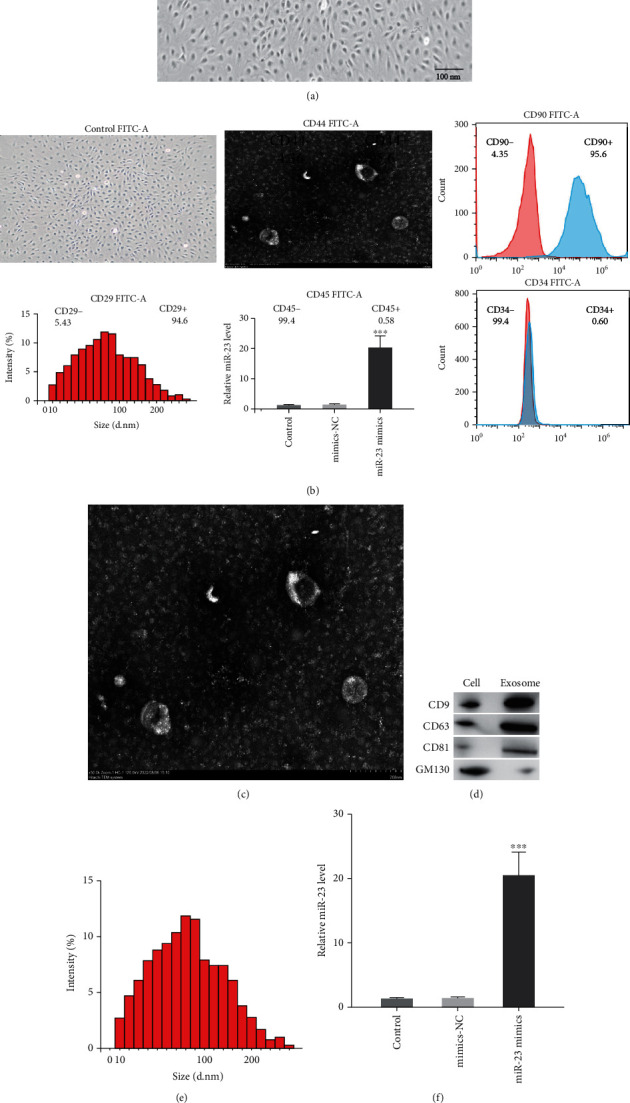
Identification of ADSC and ADSC-derived exosomes. (a) Transmission electron microscopy image of ADSC. (b) Assessment of cell surface markers of ADSC by flow cytometry. (c) Transmission electron microscopy image of ADSC-derived exosomes. (d) Western blots used to verify CD9, CD63, and TSG101 as markers of ADSC-derived exosomes. (e) Nanoparticle tracking analysis was conducted to identify the exosomes. (f) Reverse transcription PCR detection showing expression of miR-23 in exosomes after transfection. (g) The transport and uptake of ADSC-derived exosomes by macrophages were observed by fluorescence microscope. Scale bars = 100 nm. Data are presented as the mean ± SD, ^∗∗∗^*P* < 0.001.

Firstly, we extracted the ADSC from rats and identified it by transmission electron microscopy (TEM) (Figure 1(a)). Then, we identified ADSC cell phenotypes by detecting cell surface markers. The results indicated that the extracted ADSCs highly expressed positive markers such as CD29, CD90, and CD44 but were negative for endothelial markers CD34 and CD45 (Figure 1(b)). ADSC-exo was successfully separated from the ADSC supernatant by chromatography. Transmission electron microscopy showed the ADSC-exos had a circular membrane structure (Figure 1(c)). Western blotting showed that ADSC-exos expressed CD9, CD63, and CD81 and low-expressed GM130 (Figure 1(d)), and NTA results showed that the average diameter of extracted exosomes was approximately 30-180 nm. The miR-23 mimics and corresponding NC were utilized to transfect ADSCs for 48 hours. The results of RT-PCR demonstrated that miR-23 mimics could upregulate the level of miR-23 in the exosomes (Figure 1(f)). The fluorescence microscope indicated that ADSC-exos could be endocytosed by macrophages (Figure 1(g)).

### 3.2. miR-23-Enriched Exosomes Could Suppress CaOx Crystal Deposition and Renal Tubular Injury

**Figure 2:**
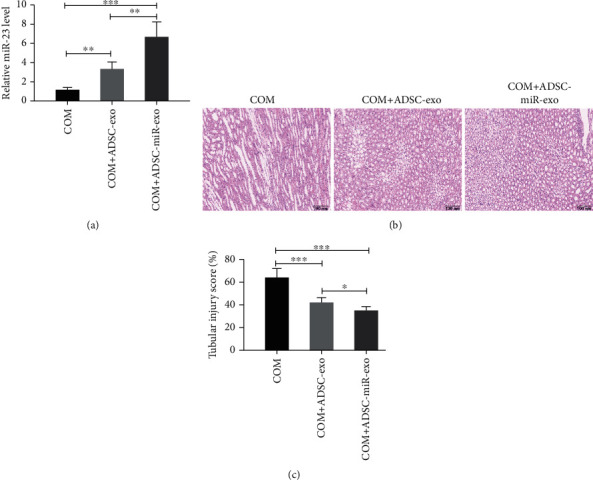
The exosomal miR-23 derived from ADSC could alleviate kidney CaOx crystal deposition and tubular injury. The exosomes from ADSC treated and untreated with miR-23 mimics were utilized to treat rats. The miR-23 expression levels in the ADSC-derived miR-23-enriched exosomes were detected by qRT-PCR (a). Then, kidney CaOx crystal deposition and tubular injury in rats were also detected (b, c). Data are presented as the mean ± SD. ^∗∗^*P* < 0.01. Scale bars = 100 nm.

The rats were sacrificed to collect kidney samples after one week of modeling. The exosomes from ADSC treated and untreated with miR-23 mimics were utilized to treat rats. The miR-23 expression levels in the ADSC-derived miR-23-enriched exosomes were detected by qRT-PCR. The results showed the exosomes from ADSC treated with miR-23 mimics could obviously elevate the miR-23 expression levels (Figure 2(a)). Besides, COM crystal deposition was detected via polarizing microscopy and Pizzolato staining. The results showed that the kidney crystals were mainly deposited in the renal cortex and medulla boundary. The number of crystals in the ADSC-derived miR-23-enriched exosome treatment group was remarkedly lower than that in the miR-23 mimics untreated group with the model group (Figure 2(b)), and the renal tubular injury scores were also significantly decreased in the treatment group (Figure 2(c)).

### 3.3. miR-23-Enriched Exosomes Could Inhibit M1 Macrophage Polarization In Vivo

**Figure 3:**
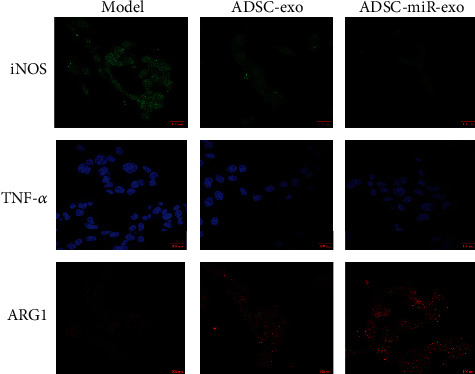
Treatment with ADSC-derived miR-23-enriched exosomes suppresses M1 macrophage polarization. Treatment with ADSC-derived miR-23-enriched exosomes could suppress iNOS and TNF-*α* expression while elevating ARG1 expression.

As shown in Figure 3, the expressions of M1-type macrophage markers iNOS and TNF-*α* were significantly increased, while the expression of M2-type macrophage maker ARG1 was obviously decreased in the calcium oxalate stone model group. In contrast, the expressions of iNOS and TNF-*α* in the treatment group were decreased, while the expression of ARG1 was elevated, especially in the ADSC-miR-exo group.

### 3.4. miR-23-Enriched Exosomes Could Alleviate COM-Induced Injury in HK-2 Cells via Repressing M1 Macrophage Polarization

**Figure 4:**
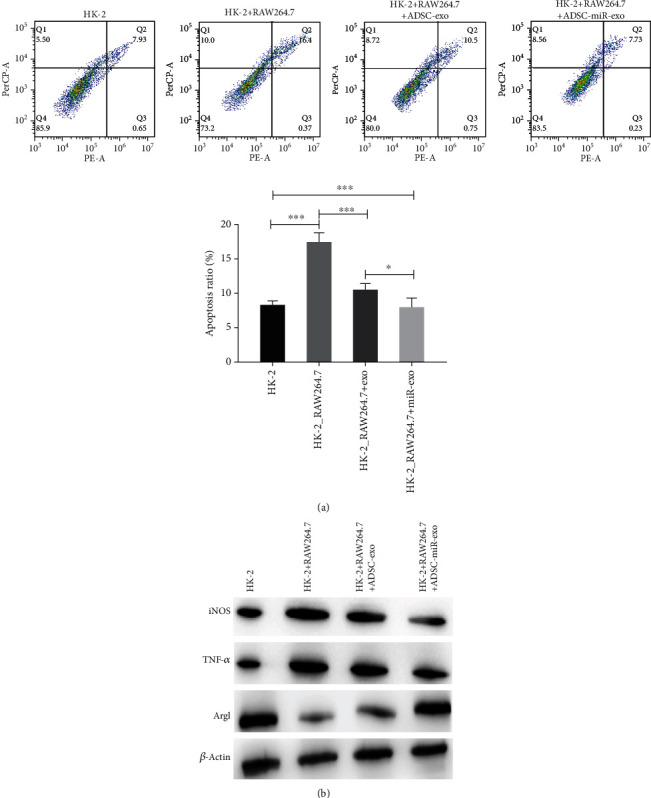
Coculturing with miR-23-enriched exosomes alleviated COM-induced injury in HK-2 cells by inhibiting M1 macrophage polarization. (a) Cell viability assessed by flow cytometry. (b) Western blotting of iNOS, TNF-*α*, and ARG1. ^∗^*P* < 0.05, ^∗∗∗^*P* < 0.001.

To determine the contribution of macrophage polarization to COM-induced kidney tubular injury, RAW264.7 cells were used to coculture with miR-23-enriched exosomes and HK-2 cells. The results illustrated that COM-induced inhibition of cell viability was obviously reversed through miR-23-enriched exosomes (Figure 4(a)), and the expression of M1 macrophage markers was remarkedly decreased in the treatment group, and M2-type macrophage maker ARG1 was obviously elevated, especially in the ADSC-miR-exo group (Figure 4(b)).

### 3.5. IRF1 Might Be the Potential Target of miR-23

**Figure 5:**
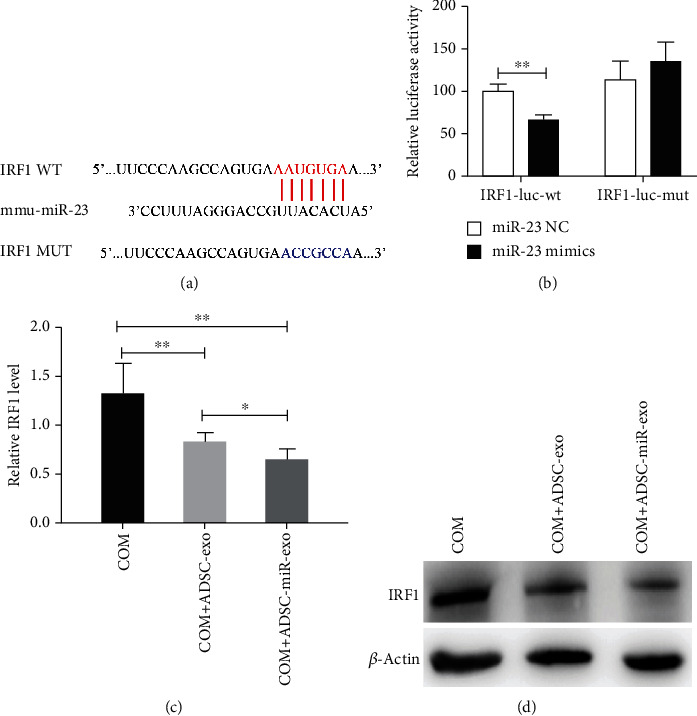
IRF1 was a target gene of miR-23. (a) The binding region between IRF1 mRNA 3′-UTR and miR-23 in wild- and mutant-type plasmids. (b) IRF1 transcriptional activity was detected by the dual-luciferase reporter gene assay system. (c, d) The expression of IRF1 was detected by RT-PCR and western blotting analysis. ^∗^*P* < 0.05, ^∗∗∗^*P* < 0.001.

To explore the biological mechanism of miR-23 on IRF1, we constructed the plasmid with WT 3′ UTR of IRF1 (IRF1-WT) or MUT 3′ UTR of IRF1 (IRF1-MUT) for further study (Figure 5(a)). Then, the plasmids were cotransfected with miR-23 mimics or NC, and a dual-luciferase reporter assay was conducted to measure the activity. The results indicated that miR-23 mimics could obviously suppress the luciferase reporter activity of the WT 3′ UTR plasmid of IRF1, but made no effect on that of IRF1-MUT (Figure 5(b)). To demonstrate the biological role of miR-23 in the polarization of M1 macrophages via IRF1, the expression level of IRF1 in RAW264.7 cells cocultured with miR-23-enriched exosomes was detected using qRT-PCR and western blot experiments. The results indicated that both the mRNA and protein levels of IRF1 were remarkably decreased in RAW264.7 cells cocultured with miR-23-enriched exosomes (Figures 5(c) and 5(d)). All the above results demonstrated that IRF1 might be the potential target of miR-23.

## 4. Discussion

Kidney stone is one of the most primarily urological diseases, with severe effects on human health and a high recurrence rate. The pathophysiological process of the formation of kidney stones involves genetic, metabolic, and environmental factors [17]. CaOx stone is the main type, accounting for approximately 70-80% of all kidney stones [2]. Therefore, exploring the mechanisms and preventions of CaOx stone formation is essential. Exosomes are lipid bilayer vesicles containing various macromolecular substances including proteins, lipids, and RNAs that can modulate intracellular signalling pathways [18]. Exosomes also participate in the regulation of numerous pathophysiological processes including immune responses, inflammation, and autophagy [19]. Recent studies have verified that exosomes are also involved in renal physiology and exert an important role in kidney stone formation [20–22]. However, exosomes' effects on treating kidney stones remain unclear. Therefore, the aim of the present study was to explore the efficacy of ADSC-derived exosomes in treating calcium oxalate kidney stones.

Previous researches have manifested that damage to the renal tubular epithelial cells caused by inflammation may promote calcium oxalate crystal adhesion and stone formation [23]. However, the underlying mechanisms remain unclear. As reported, miR-23 can regulate inflammatory responses [24]. For example, increased expression of miR-23 in serum is associated with renal damage in early lupus nephritis, whereas overexpression of miR-23 exhibits an ameliorating effect on lupus nephritis via suppressing IRF1 in vivo [6]. IRFs exert a vital role in modulating the polarization of macrophages. Accumulating evidence has demonstrated that IRF1 participates in the commitment of M1 macrophages through activating and inhibiting the transcription of targets [25]. In this study, we found decreased expression of M1-type macrophage markers in the miR-23-enriched ADSC-derived exosome treatment group, which revealed that miR-23 might have a vital effect on inflammation and M1 macrophage polarization. The results of dual-luciferase reporter assays showed that IRF1 might be the direct target of miR-23, and miR-23-enriched exosomes suppress M1 polarization by inhibiting IRF1. In vivo experiments showed that treatment with ADSC-derived miR-23-enriched exosomes could repress CaOx crystal deposition and renal tubular injury. In vitro experiments also revealed that COM-induced suppression of cell viability was remarkedly reversed by coculturing with miR-23-enriched exosomes. Our study verified that ADSC-derived miR-23-enriched exosomes inhibited COM-induced inflammation via inhibiting IRF1 expression. These results indicated the protective effects of ADSC-derived miR-23-enriched exosomes on calcium oxalate stone formation.

Overall, our study explored and revealed that treatment with ADSC-derived miR-23-enriched exosomes might have an effective therapeutic effect on calcium oxalate kidney stones. It might provide a novel insight into the molecular mechanisms and therapeutic effects of ADSC-derived miR-23-enriched exosomes in treating kidney stones.

## Data Availability

The data that support the findings of this study are available from the corresponding authors (xlw1980@126.com, ruipengj@163.com) upon reasonable request.
